# Supplementary figures and images for: A novel SATB1 protein isoform with different biophysical properties
Source: Front Cell Dev Biol. 2023 Aug 11;11:1242481. doi: 10.3389/fcell.2023.1242481 (PMC10457122; doi:10.3389/fcell.2023.1242481)

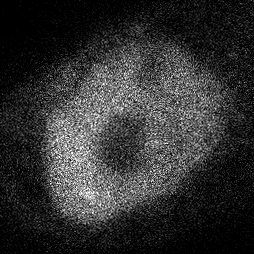

Supplement: Supplementary file 4 [file DataSheet5.zip › SupplementaryFile5_optoDroplet_assembly/CRY2-mCherry_SATB1-N-terminus.gif]

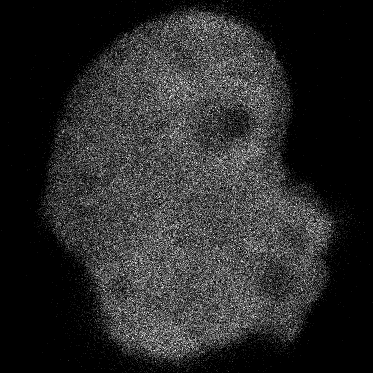

Supplement: Supplementary file 4 [file DataSheet5.zip › SupplementaryFile5_optoDroplet_assembly/CRY2-mCherry_FUS-N-terminus.gif]
